# SenoIndex: S100A8/S100A9 as a novel aging biomarker

**DOI:** 10.1093/lifemedi/lnad022

**Published:** 2023-06-13

**Authors:** Baohu Zhang, Haoteng Yan, Xiaoqian Liu, Liang Sun, Shuai Ma, Si Wang, Jing Qu, Guang-Hui Liu, Weiqi Zhang

**Affiliations:** State Key Laboratory of Stem Cell and Reproductive Biology, Institute of Zoology, Chinese Academy of Sciences, Beijing 100101, China; University of Chinese Academy of Sciences, Beijing 100049, China; Advanced Innovation Center for Human Brain Protection, and National Clinical Research Center for Geriatric Disorders, Xuanwu Hospital Capital Medical University, Beijing 100053, China; Aging Translational Medicine Center, Xuanwu Hospital, Capital Medical University, Beijing 100053, China; State Key Laboratory of Stem Cell and Reproductive Biology, Institute of Zoology, Chinese Academy of Sciences, Beijing 100101, China; University of Chinese Academy of Sciences, Beijing 100049, China; Institute for Stem Cell and Regeneration, Chinese Academy of Sciences, Beijing 100101, China; Beijing Institute for Stem Cell and Regenerative Medicine, Beijing 100101, China; NHC Beijing Institute of Geriatrics, NHC Key Laboratory of Geriatrics, Institute of Geriatric Medicine of Chinese Academy of Medical Sciences, National Center of Gerontology/Beijing Hospital, Beijing 100730, China; Institute for Stem Cell and Regeneration, Chinese Academy of Sciences, Beijing 100101, China; Beijing Institute for Stem Cell and Regenerative Medicine, Beijing 100101, China; Aging Biomarker Consortium, Beijing 100101, China; State Key Laboratory of Membrane Biology, Institute of Zoology, Chinese Academy of Sciences, Beijing 100101, China; Advanced Innovation Center for Human Brain Protection, and National Clinical Research Center for Geriatric Disorders, Xuanwu Hospital Capital Medical University, Beijing 100053, China; Aging Translational Medicine Center, Xuanwu Hospital, Capital Medical University, Beijing 100053, China; Aging Biomarker Consortium, Beijing 100101, China; State Key Laboratory of Stem Cell and Reproductive Biology, Institute of Zoology, Chinese Academy of Sciences, Beijing 100101, China; University of Chinese Academy of Sciences, Beijing 100049, China; Institute for Stem Cell and Regeneration, Chinese Academy of Sciences, Beijing 100101, China; Beijing Institute for Stem Cell and Regenerative Medicine, Beijing 100101, China; Aging Biomarker Consortium, Beijing 100101, China; University of Chinese Academy of Sciences, Beijing 100049, China; Advanced Innovation Center for Human Brain Protection, and National Clinical Research Center for Geriatric Disorders, Xuanwu Hospital Capital Medical University, Beijing 100053, China; Institute for Stem Cell and Regeneration, Chinese Academy of Sciences, Beijing 100101, China; Beijing Institute for Stem Cell and Regenerative Medicine, Beijing 100101, China; Aging Biomarker Consortium, Beijing 100101, China; State Key Laboratory of Membrane Biology, Institute of Zoology, Chinese Academy of Sciences, Beijing 100101, China; University of Chinese Academy of Sciences, Beijing 100049, China; Institute for Stem Cell and Regeneration, Chinese Academy of Sciences, Beijing 100101, China; Aging Biomarker Consortium, Beijing 100101, China; CAS Key Laboratory of Genomic and Precision Medicine, Beijing Institute of Genomics, Chinese Academy of Sciences, Beijing 100101, China; China National Center for Bioinformation, Beijing 100101, China

**Dear Editor,**

Aging is associated with progressive physiological decline and biological dysfunction, which is therefore considered a major risk factor for age-related chronic diseases, such as cardiovascular diseases (CVD), neurodegenerative diseases, and cancer [1]. Due to the complexity of the aging process, biomarkers that can identify the degree of aging are needed [2]. Through in-depth explorations, age-related molecular mechanisms have been discovered, such as stimulation of senescence-associated secretory phenotype (SASP), telomere attrition, and epigenetic alterations [3, 4]. However, to more fully understand how to mitigate the aging process and identify preventive measures in age-related chronic diseases, robust aging biomarkers that can be used to evaluate aging status are needed.

With the rapid development and the advantages of single-cell/nucleus RNA-sequencing (scRNA-seq/snRNA-seq) technologies over the past decade, we have been able to explore cell heterogeneity during the aging processes at transcriptional levels [5, 6]. Recently, scientists have tried to understand the heterogeneity during aging at molecular and cellular levels for each type of organ in certain species, which brings opportunities for discovering new aging biomarkers. Extending from the abovementioned findings, the identification of biomarkers capable of assessing the extent of aging across various cell types, tissues, and species, especially those detectable in peripheral blood, is of paramount importance for current investigations. In this study, by integrating publicly available single-cell sequencing datasets from the Aging Atlas (https://ngdc.cncb.ac.cn/aging/index) [7], we identified S100 calcium-binding protein A8 (S100A8) and S100A9, which belong to the S100 gene family, as the most frequently upregulated genes in various cell types across species. Furthermore, we confirmed that heterodimer of S100A8 and S100A9 (S100A8/S100A9) was increased in aged tissues of different species. In addition, S100A8/S100A9 accrues in human serum with aging, suggesting that S100A8/S100A9 may serve as a potential biomarker for assessing human aging. In addition, we found that cells underwent accelerated senescence upon treatment with recombinant human S100A8/S100A9 protein (rhS100A8/S100A9), demonstrating its potential aging driving role.

We previously constructed a rat single-cell transcriptional landscape to study the mechanism of aging and its intervention by caloric restriction at the systemic level [8]. Here, we re-analyzed differentially expressed genes (DEGs) in 9 tissues and 118 cell types from this study and identified the top frequently changed genes encoding secreted proteins that varied with age (Fig. S1A and S1B). Compared with young rats, the top five upregulated genes in aged rats were *S100a8*, *S100a9*, *Il1b*, *Apoe*, and *Anxa1*, and the top five downregulated genes were *Sparc*, *Ybx1*, *Igfbp7*, *Apoc1*, and *Igfbp6* (Fig. S1B). Among these genes, *Il1b*, encoding IL1β, a widely recognized indicator of inflammatory cytokine known as SASP, exhibits elevated levels with advancing age. This upregulation suggests the presence of accumulated senescent cells and heightened chronic inflammation within tissues, particularly in the brain, liver, and pancreatic islets. Notably, scRNA-seq data showed that *S100a8* and *S100a9* were upregulated in multiple tissues and organs of aged rats, even with a higher frequency than *Il1b* (Fig. S1B).

To identify aging biomarkers across different organism models, including *Rattus norvegicus*, *Mus musculus*, *Macaca fascicularis*, and *Homo sapiens*, we collected more than 100,000 aging-associated differentially expressed genes (aging DEGs) based on the scRNA-seq and snRNA-seq datasets archived in Aging Atlas (Fig. 1A and 1B). To screen Aging Hub Genes (AHGs), we utilized the protein-protein interaction (PPI) network to predict the interaction across all the aging DEGs, and then we ranked these genes according to their gene affinity score that defines the network vicinity of each gene by a Random Walk with Restart (RWR) algorithm (See Materials and methods). Through the employed approach, we identified a comprehensive set of 9,539 candidate AHGs across 4 species, 41 tissues, and 444 cell types. Among these candidate AHGs, a subset of 3,146 genes displayed potential interactions exclusively with upregulated aging DEGs (referred to as up-specific candidate AHGs). Another subset of 4,178 genes demonstrated potential interactions with downregulated aging DEGs (referred to as down-specific candidate AHGs). Additionally, we observed that 2,215 candidate AHGs exhibited potential interactions with both upregulated and downregulated aging DEGs simultaneously (referred to as shared candidate AHGs). These data suggested the specific involvement of candidate AHGs as central nodes in the intricate regulatory network underlying aging (Fig. 1C and 1D, See Methods). The up-specific candidate AHGs were enriched in terms of the inflammatory response and aging (Fig. 1E), and the down-specific candidate AHGs were enriched in terms of the regulation of growth and regeneration (Fig. 1F), in agreement with the importance of these pathways in regulating aging as reported previously [8]. The subset of shared candidate AHGs identified in our study exhibited participation in processes associated with protein synthesis and secretion (Fig. 1G), highlighting the potential of secretory proteins as valuable aging biomarkers.

**Figure 1.**
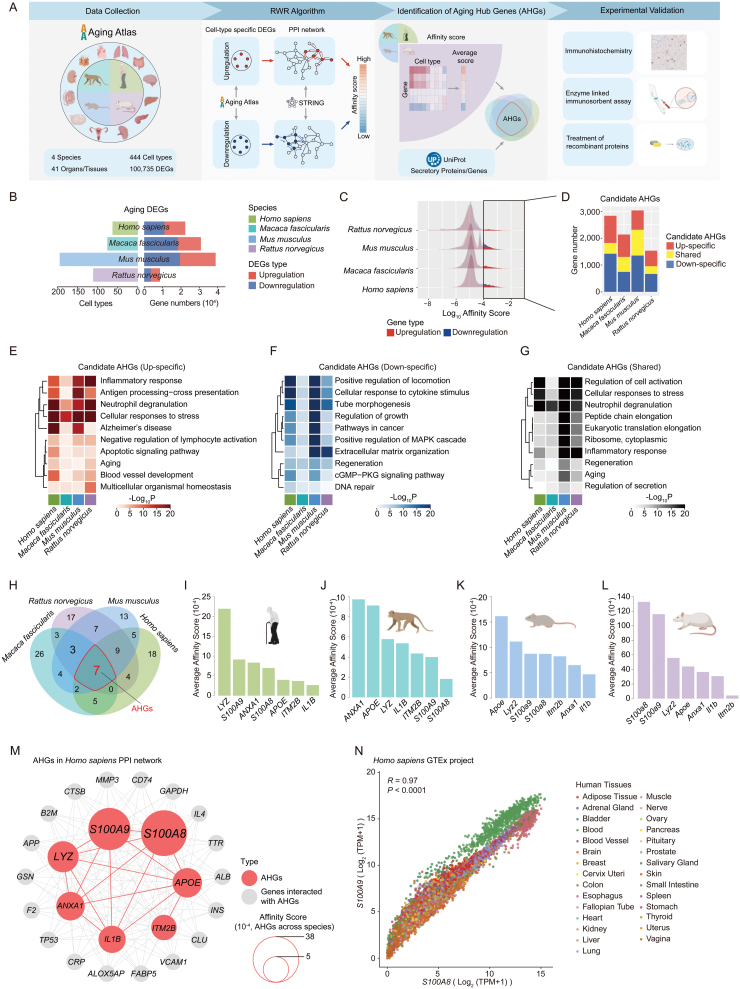
**Identification of potential aging biomarkers based on single-cell transcriptome data.** (A) The process diagram showing that the data sets from Aging Atlas were used for identification of AHGs and then validated experimentally. (B) Bar plots showing the number of cell types (left panel) and genes (right panel) of Aging Atlas used for analysis. (C–D) The affinity score distribution (C) and gene number (D) of candidate AHGs across species. Candidate AHGs were defined as Gene Affinity Score ≥ 1 × 10^−4^. (E–G) The enrichment analysis of candidate AHGs from up-specific (E), down-specific (F) and shared candidate AHGs (G). (H) The identification of AHGs was performed by ranking the candidate AHGs that encode secretory proteins with affinity score identified by the RWR algorithm. Seven genes were highlighted by overlapping the top 50 candidate AHGs encoding secretory proteins of each species as AHGs. (I–L) Bar plot showing the affinity score of the seven AHGs across *Homo sapiens* (I)*, Macaca fascicularis* (J)*, Mus musculus* (K), and *Rattus norvegicus* (L). (M) The human protein-protein interaction network of the seven AHGs. Genes with higher affinity score are larger in size. (N) The scatter plot showing mRNA expression levels of *S100A8* and *S100A9* across 29 human tissues from the GTEx project.

Among candidate AHGs, genes encoding secretory proteins account for 3% to 9% (Fig. S1C). These genes hold the potential as aging biomarkers. Hence, candidate AHGs encoding secretory proteins were selected for ranking by averaged affinity score across species. Notably, the top 50 candidate AHGs encoding secretory proteins were enriched in convergent biological pathways, such as inflammatory response, leukocyte chemotaxis, cell chemotaxis, and vascular morphogenesis (Figs. 1H and S1D–E). In particular, seven overlapped top candidate AHGs encoding secreted proteins overlapping in all species were highlighted and defined as AHGs, including *S100A9*, *S100A8*, *ITM2B*, *IL1B*, *APOE*, *LYZ*, and *ANXA1* (Fig. 1I–L; Table S1). Among the seven AHGs, *S100A9* and *S100A8* had the highest average affinity score (Fig. 1M). In addition, data from the GTEx project showed a strong correlation score between *S100A8* and *S100A9* at the transcriptional level across human tissues (Fig. 1N). Taken together, these findings suggested that S100A8 and S100A9 may harmonically indicate the extent of aging across both species and tissues.

We then performed a series of experiments to verify the increased expression of S100A8 and S100A9 in various rodent and nonhuman primate tissues during physiological aging. We found an intensified expression of S100A8 in the kidney, lung, liver, and brain of aged (24-month-old) mice relative to young (4-month-old) mice (Figs. 2A and S2A). The immunohistochemical staining also revealed that S100A8 and S100A9 accumulated in the skin, kidney, lung, liver, and brain tissue of physiologically aged (18–21 years old, similar to 65–70 human age) cynomolgus monkeys compared with young (4–6 years old, similar to 16–20 human age) cynomolgus monkeys (Figs. 2B–C and S2B–D). Previous studies indicated that S100A8 and S100A9 might exist as heterodimers and cooperatively exert *in vivo* biological functions [9]. We then wondered whether the abundance of S100A8/S100A9 in peripheral blood was also augmented with age in humans, and to answer that question, we collected healthy human serum samples of different age groups (Fig. 2D). Through ELISA analysis, we detected increased levels of the S100A8/S100A9 (2.36-Fold, *P* < 0.001) in the serum of the elderly (Fig. 2E). Taken together, our results highlight the role of S100A8/S100A9 as a potential aging biomarker for assessing human aging at both tissue and organismal levels.

**Figure 2.**
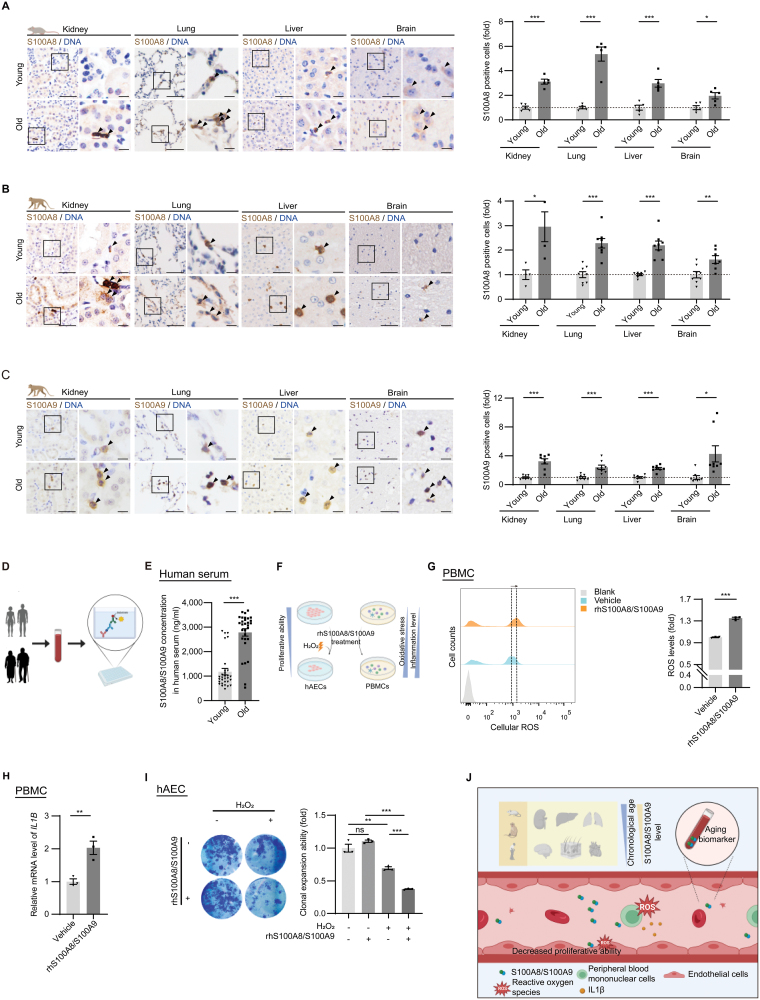
**S100A8/S100A9 functions as both a biomarker and a driver of aging.** (A) Representative immunohistochemistry of S100A8 in kidney, lung, liver, and brain of young and old mice with higher magnification of indicated region shown at right. Scale bars, 25 μm and 5 μm (zoomed-in image). Data are shown as means ± SEM. *n* = 5, number of animals. *P < 0.05, ***P < 0.001, (*t* test). (B) Representative immunohistochemistry of S100A8 in kidney (*n* = 4), lung (*n* = 8), liver (*n* = 8) and brain (*n* = 7 or 8) of young and old cynomolgus monkeys with higher magnification of indicated region shown at right. Scale bars, 25 μm and 5 μm (zoomed-in image). Data are shown as means ± SEM. *n*, number of animals. *P < 0.05, **P < 0.01, ***P < 0.001, (*t* test). (C) Representative immunohistochemistry of S100A9 in kidney (*n* = 8), lung (*n* = 8), liver (*n* = 8), and brain (*n* = 8) of young and old cynomolgus monkeys with higher magnification of indicated region shown at right. Scale bars, 25 μm and 5 μm (zoomed-in image). Data are shown as means ± SEM. *n*, number of animals. *P < 0.05, ***P < 0.001, (*t* test). (D) Schematic diagram showing the experimental procedure for the ELISA analysis. (E) ELISA analysis of S100A8/S100A9 levels in serum from young and old people. *n* = 30, biological samples. ***P < 0.001, (*t* test). (F) Schematic diagram showing the experimental procedure for G, H and I. (G) FACS measurement of ROS levels by staining PBMCs with the probe H2DCFDA after treatment with rhS100A8/S100A9 (10 μg/mL). Data are shown as means ± SEM*. n =* 3, biological replicates. ***P < 0.001, (*t* test). (H) RT-qPCR analysis of *IL1B* in PBMCs after treatment with rhS100A8/S100A9. Data are shown as means ± SEM. *n* = 3 biological replicates. **P < 0.01, (*t* test). (I) Clonal expansion assay of hAECs after co-treatment with rhS100A8/S100A9 (2 μg/mL) and H_2_O_2_ (100 μM). Data are shown as means ± SEM. *n =* 3, biological replicates. ns, non-significant, **P < 0.01, ***P < 0.001, (*t* test). (J) Schematic diagram of S100A8/S100A9 as a novel aging biomarker.

In previous studies, S100A8/S100A9 was reported to be secreted into the extracellular space during acute or chronic inflammation, where it acted as a proinflammatory damage-associated molecular pattern (DAMP) [9]. To further decipher the physiological impacts of accumulated S100A8/S100A9 during aging, we treated human peripheral blood mononuclear cells (PBMCs) with rhS100A8/S100A9 (Fig. 2F). We found that rhS100A8/S100A9 treatment led to an elevation of reactive oxide species (ROS) levels (Fig. 2G), and an increased expression of the proinflammatory factor *IL1B* (Fig. 2H) in PMBCs, indicating that augmented S100A8/S100A9 in the serum could disrupt the homeostasis of blood cells in the elderly. Moreover, rhS100A8/S100A9 supplementation compromised the proliferative ability of human aortic endothelial cells (hAECs), as manifested by the decrease of Ki67-positive cells and declined clonal expansion ability under the condition of oxidative stress (Figs. 2I and S2E). Hence, the increased S100A8/S100A9 abundance might elicit oxidative stress, inflammatory response, and proliferative impairment In a variety of vascular and blood-associated cell types. Conversely, targeting or blocking S100A8/S100A9 may represent a potential therapeutic strategy for alleviating cellular senescence and tissue degeneration, but this requires a massive of *in vitro* and *in vivo* experiments to verify.

In this study, we leveraged comprehensive bioinformatic analysis of large datasets and multi-modal experiments to identify S100A8/S100A9 as a novel aging biomarker that is increased in multiple aged tissues across species. Moreover, our data show that S100A8/S100A9 triggers increases in intracellular ROS levels and promotes the transcription of the proinflammatory cytokine *IL1B* in PBMCs. Moreover, elevated levels of serum S100A8/S100A9 in the elderly also demonstrate the potential of S100A8/S100A9 as a serum biomarker for clinical assessment of aging and provide a potential target for aging intervention strategies (Fig. 2J).

In previous studies, S100A8/S100A9 was found to play a proinflammatory role in numerous inflammatory diseases, mediating leukocyte recruitment, and inducing the expression of proinflammatory factors and cytokines [9, 10]. Consistently, a prolonged proinflammatory state known as low-grade sterile chronic inflammation is widely recognized as a major driver of aging [2]. Previous studies have shown that S100A8/S100A9 is elevated in age-related diseases, including atherosclerosis and Alzheimer’s disease (AD) [9]. Our results demonstrated that S100A8/S100A9 can promote the expression of proinflammatory cytokines *IL1B*, and contributes to the upregulation of intracellular levels of ROS. Mechanistically, continuous secretion of proinflammatory factors maintains the proinflammatory cell state and transmits senescence signals to neighboring cells, while a sustained rise in intracellular ROS leads to oxidative stress and exacerbates cellular senescence. All these results suggest that S100A8/S100A9 might not only serve as a novel aging biomarker, but also as a potential aging driver. Simultaneously, our dataset also found several extra AHGs encoding secreted proteins that can serve as potential aging biomarkers, including *ITM2B*, *IL1B*, *APOE*, *LYZ*, and *ANXA1*. Among them, *APOE*, encoding apolipoprotein E, is a major genetic risk factor for late-onset AD and is also associated with CVD and longevity. The high level of IL1β leads to cellular senescence and decreased function of tissues. The Integral membrane protein 2B encoded by *ITMB2* has been reported to interact with the amyloid precursor protein and influences the production of amyloid beta peptides. Meanwhile, a study on the brains of AD patients showed that the integral membrane protein 2B accumulates in amyloid plaques. However, further investigation is required to fully explore the potential of these candidates as biomarkers of aging.

We are sparing no effort to establish the “SenoIndex”, a comprehensive framework that will encompass a broader range of individual aging biomarkers, including those that yet to be discovered. Our focus is particularly on identifying biomarkers present in bodily fluid such as human plasma. The SenoIndex will help reveal the underpinnings of the complex processes of aging, laying the foundation for analyzing aging characteristics. As such, it will not only provide a reference for exploring the molecular mechanism of aging, but will also serve as a reference for clinical trial design to improve human healthy life expectancy.

## Research limitations

Although we demonstrated increased expression of S100A8/S100A9 in aging tissues and peripheral blood, we currently lack substantial evidence regarding age-dependent alteration in S100A8/S100A9 within a large cohort. At the same time, *in vivo* experiments of S100A8/S100A9 neutralizing antibodies or chemical inhibitors can strengthen our view more powerfully, which is also what we need to pay attention to, and the upregulation of S100A8/S100A9 during aging across tissues requires us to establish more senescent cell models. More efforts are needed to reveal the role of S100A8/S100A9 in aging promotion and aging intervention.

## Supplementary Material

lnad022_suppl_Supplementary_Figures

lnad022_suppl_Supplementary_Material

lnad022_suppl_Supplementary_Tables
