# Supplementary figures and images for: SenoIndex: S100A8/S100A9 as a novel aging biomarker
Source: Life Med. 2023 Jun 13;2(4):lnad022. doi: 10.1093/lifemedi/lnad022 (PMC11749476; doi:10.1093/lifemedi/lnad022)

Figure S1

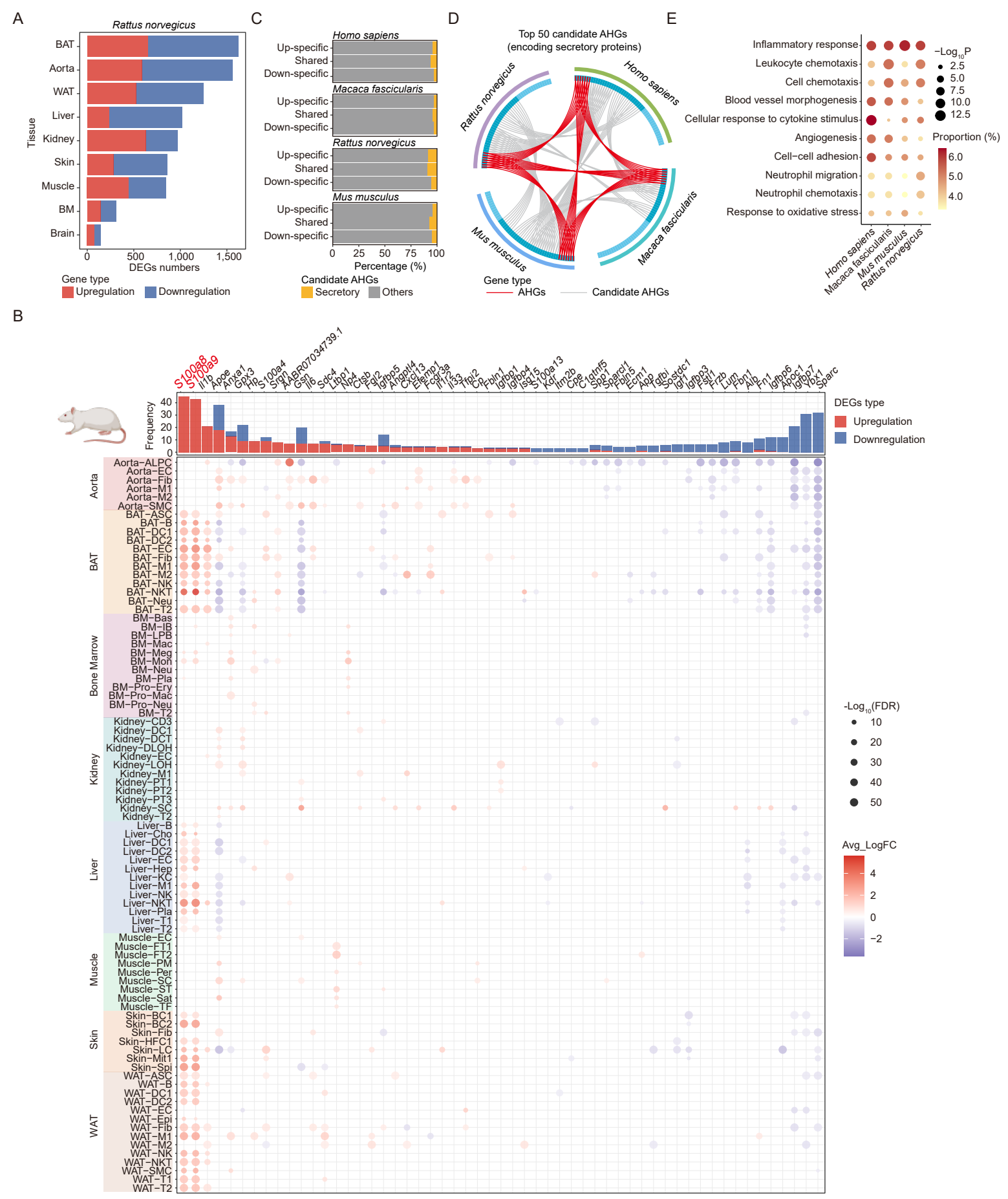

Figure S2

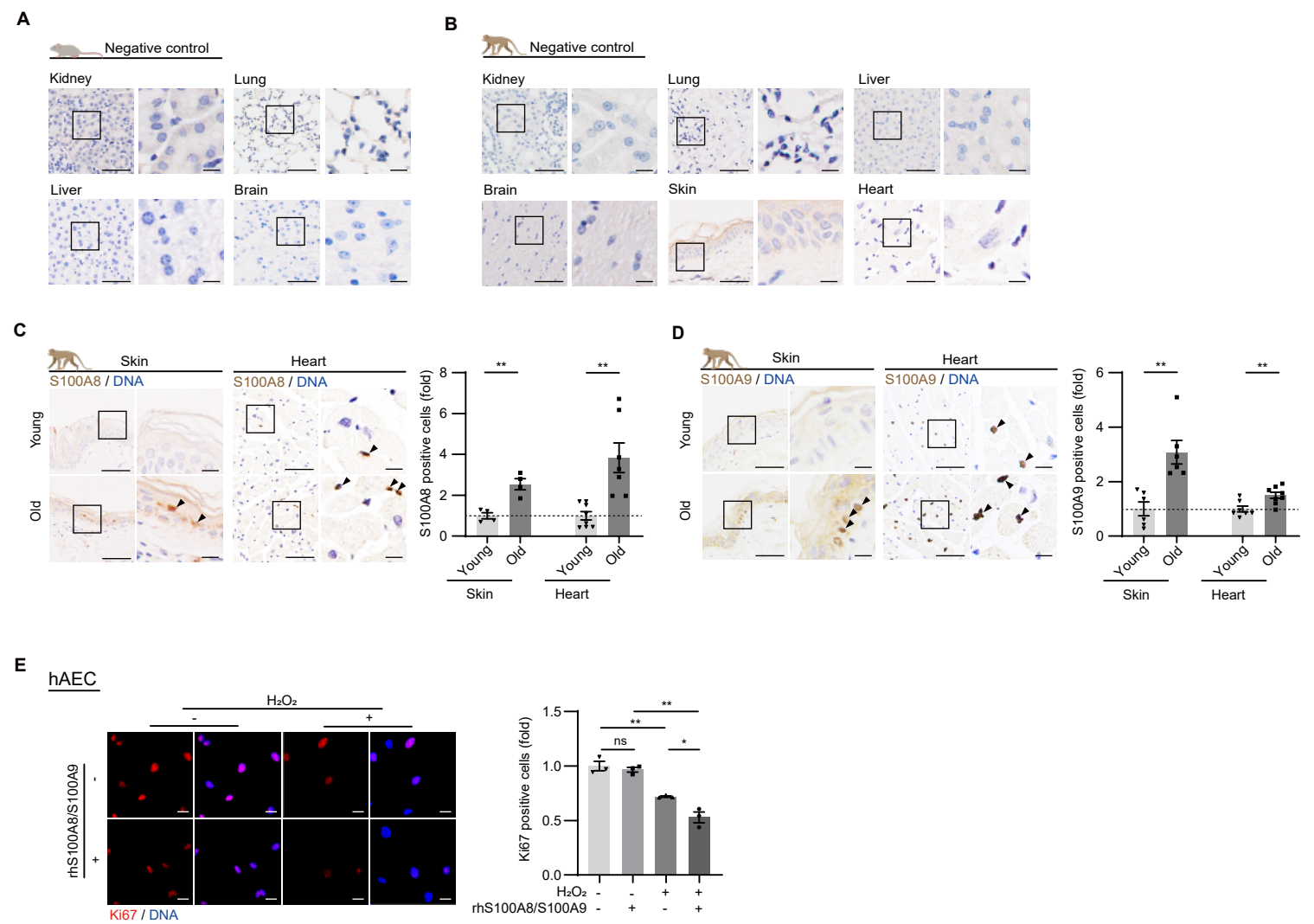

Supplement: lnad022_suppl_Supplementary_Figures [file lnad022_suppl_Supplementary_Figures.pdf]
