# Supplementary material for: SenoIndex: S100A8/S100A9 as a novel aging biomarker
Source: Life Med. 2023 Jun 13;2(4):lnad022. doi: 10.1093/lifemedi/lnad022 (PMC11749476; doi:10.1093/lifemedi/lnad022)
Supplement: lnad022_suppl_Supplementary_Material [file lnad022_suppl_Supplementary_Material.docx]

**Supplementary Information**

**SenoIndex: S100A8/S100A9 as** **a novel aging biomarker**

**Materials and Methods**

**Identification of aging hub genes**

To identify Aging Hub Genes (AHGs) across the four species, including *Homo sapiens*, *Macaca fascicularis*, *Rattus norvegicus* and *Mus musculus*, we downloaded and authorized for further analysis the differentially expressed genes (DEGs) of sc/snRNA-seq datasets during organs aging from Aging Atlas database. Combined with the Protein-Protein Interaction (PPI) network from the STRING database [1], we leveraged the Random Walk with Restart (RWR) algorithm in R package dnet V1.1.7 across species and cell types to calculate the affinity score of each gene, which reflected the influence of the gene on the entire PPI network [2]. The upregulated DEGs and downregulated DEGs of each cell type were used as two different groups of seed genes to perform RWR respectively, and the average score was used as the affinity score of each cell type. The candidate AHGs were defined as genes with an affinity score ≥ 1×10^-4^ in both upregulated and downregulated genes. The average affinity score of upregulated and downregulated gene scores was calculated as the final affinity score across organs or species. Then, each gene in the PPI network had an affinity score. The AHGs were defined as genes overlapping across the four species from the top 50 candidate AHGs (encoding secretory proteins) annotated by the UniProt database, where the genes of non-human species were annotated as human genes through homologous genes in the Ensembl gene annotation system.

**Experimental animals and ethics**

Mice experiments were approved by the Ethics Review Committee of the Institute of Zoology, Chinese Academy of Sciences. C57BL/6 J male mice purchased from SiPeiFu (Beijing) Biotechnology Co., Ltd were raised at 25°C in a 12-hr light-dark cycle in the animal care facility at the Institute of Zoology, Chinese Academy of Sciences. Euthanization of mice was performed with CO_2_ followed by cervical dislocation. And the tissues were collected. The cynomolgus monkey experiments were conducted in accordance with the Ethical Treatment Principles of Non-Human Primates and experiments were approved by the Animal Protection and Ethics Committee of Institute of Zoology, Chinese Academy of Sciences. Both young and old cynomolgus monkeys used in the experiments were raised at the Xieerxin Biology Resource, which is certified by the Beijing Laboratory Animal Protection And Certification Authority, in compliance with all local and federal laws governing animal research. The lung, liver, kidney, brain, heart and skin samples were collected from 8 young and 8 old cynomolgus monkeys without clinical or experimental history. The cynomolgus monkeys used in this study were the same ones reported in the previous studies [3]. Human serum samples were collected with the approval of the Research Ethics Committee of the First Hospital of Kunming Medical University, and details were provided in Table S2. The acquisition of human PBMCs was approved by the West China Fourth Hospital of Sichuan University.

**Cell culture**

Human arterial endothelial cells (HAECs) were grown on collagen (Sigma)-coated plates (CORNING) in EGM2 medium (Lonza) supplemented with 1% penicillin/streptomycin (Gibco). Human PBMCs were cultured in RPMI 1640 (Gibco) supplemented with 10% FBS (Gibco), 2 mmol/L GlutaMAX (Gibco), 0.1 mmol/L NEEA (Gibco), 1 mmol/L Pyruvic acid sodium (Gibco) and 1% penicillin/streptomycin. Cells were cultured in an incubator (Thermo Fisher Scientific) at 37°C with 5% CO_2_. No mycoplasma contamination was observed during cell culture.

**Cell stimulation with recombinant protein**

HAECs were grown in EGM2 medium till 80% confluency, and then cultured for 24 hr either in EGM2 medium supplemented with vehicle or 2 µg/mL recombinant human S100A8/S100A9 protein (rhS100A8/S100A9) (Sino Biological). Human PBMCs were cultured for 24 hr in RPMI 1640 medium supplemented with vehicle or 10 µg/mL rhS100A8/S100A9.

**Immunofluorescence, immunohistochemistry staining and microscopy**

For immunofluorescence, cells cultured on coverslips (Thermo Fisher Scientiﬁc) were washed twice with PBS, fixed in 4% PFA for 20 min, permeabilized with 0.4% TritonX-100 (Sigma) for 20 min, and blocked for 1 hr at room temperature with 5% donkey serum (Jackson Immuno Research) diluted with PBS. Subsequently, the cells were incubated with primary antibody at 4°C overnight. Then the cells were incubated with secondary antibody at room temperature for 1 hr, and nuclei were stained with Hoechst 33342 (Thermo Fisher Scientific). Immunohistochemistry staining of tissue sections was performed using the DAB Staining Kit (ZSGB-BIO) according to the manufacturer’s instructions. Briefly, the sections were deparaffinized and rehydrated. Endogenous peroxidase activity was quenched by incubation with 3% hydrogen peroxide for 20 min before antigen retrieval using 10 mM sodium citrate buffer (pH 6.0). Sections were then permeabilized, blocked and incubated with primary antibodies at 4°C overnight. Slides were then washed three times with PBS and incubated with secondary antibodies for 1 hr at room temperature. After three washes with PBS, the sections were incubated with DAB chromogen and counterstained with hematoxylin. Images were taken using the ZEISS LSM900 confocal microscope or PerkinElmer Vectra Polaris. The primary antibody used for immunofluorescence were anti-Ki67 (ZM-0166, 1:500) from ZSGB-Bio and the secondary antibody was Alexa 568 donkey anti-mouse IgG (A10037, 1:500) from Thermo Fisher Scientific. The primary antibodies used for immunohistochemistry were anti-S100A8 (ab92331, 1:400) and anti-S100A9 (ab92507, 1:400) from abcam.

**Flow cytometry analysis**

For detection of total intracellular ROS, living cells were incubated with CM-H2DCFDA (C6827, Invitrogen) for 30 min at room temperature. Incubated cells were performed using the LSR Fortessa cell analyzer (BD), and the data were analyzed with the FlowJo software (TreeStar, Ashland, OR).

**Clonal expansion assay**

Two thousand cells were seeded in one well of a 12-well plate precoated with gelatin and cultured for approximately 10 days. The cells were fixed with 4% PFA for 30 min and stained with 10% crystal violet for 30 min. The relative cell integral density was calculated by ImageJ software.

**Quantitative real-time PCR (RT-qPCR)**

Total RNA from cells or tissues was extracted by TRIzol (15596018, Gibco) and reverse-transcribed into cDNA using GoScript Reverse Transcription System (A5001, Promega). RT-qPCR was then performed using SYBR qPCR mix (QPS-201, TOYOBO) on a CFX384-Real-time system (Bio-Rad). The primers used for RT-qPCR are listed in Table S3.

**Enzyme linked immunosorbent assay (ELISA)**

For young and old serum samples, S100A8/S100A9 levels were determined using a commercial ELISA kit (R&D Systems) according to the manufacturer’s instructions. In brief, 100 µL human serum (Dilute 500 times) was added into the pre-prepared coating plate and incubated for 2 hr at room temperature. After incubation with Detection Antibody, Streptavidin-HRP, Substrate Solution and Stop Solution. Then, plates were measured at 450 nm by using the Synergy H1 Microplate Reader (BioTek).

**Statistical analysis**

Statistical analyses were performed using the Prism version 8 software (GraphPad Software). Data were presented as means ± SEM. Comparisons were made by the two-tailed student's *t* test. *P* values < 0.05 were considered statistically significant (*), *P* values < 0.01 were considered highly statistically significant (**) and *P* values < 0.001 were considered highly statistically significant (***).

**Data availability**

The data supporting the findings of this study are available within the article or its supplementary materials.

**References in methods**

1. Szklarczyk, D., et al., *The STRING database in 2021: customizable protein-protein networks, and functional characterization of user-uploaded gene/measurement sets.* Nucleic Acids Res, 2021. 49(D1): p. D605-D612.

2. Fang, H. and J. Gough, *The 'dnet' approach promotes emerging research on cancer patient survival.* Genome Med, 2014. 6(8): p. 64.

3. Zhang, H., et al., *Single-nucleus transcriptomic landscape of primate hippocampal aging.* Protein Cell, 2021. 12(9): p. 695-716.

**Supplemental figure legends**

**Figure S1. Idenfication of aging hub genes based on the aging DEGs of rats and other species.**

(A) Bar plot showing the number of aging DEGs that encode secretory proteins during *Rattus Norvegicus* aging at single cell level. (B) The top 30 upregulated genes and top 30 downregulated genes encoding secretory proteins with the highest frequency during *Rattus Norvegicus* aging at single cell level. The top panel is the frequency of the top genes. The bottom panel is the fold change and *P* value of the top genes across various tissues and cell types. (C) Bar plot showing the percentage of secretory genes in candidate AHGs across species. (D) The plot showing the top 50 candidate AHGs that encode secretory proteins ranked by affinity score across *Rattus norvegicus*, *Mus musculus*, *Macaca fascicularis* and *Homo sapiens*, respectively. (E) Enriched GO terms for the top 50 candidate AHGs that encode secretory proteins ranked by affinity score across *Rattus norvegicus*, *Mus musculus*, *Macaca fascicularis* and *Homo sapiens*, respectively. The proportion indicates the percentage of annotated genes in all genes among each GO term.

**Figure S2. Detection of the levels and effects of S100A8/9 during aging.**

(A) Representative immunohistochemistry images of negative control staining of mice with higher magnification of indicated region shown at right. Scale bars, 25 μm and 5 μm (zoomed-in image). (B) Representative immunohistochemistry images of negative control staining of cynomolgus monkeys with higher magnification of indicated region shown at right. Scale bars, 25 μm and 5 μm (zoomed-in image). (C) Representative immunohistochemistry images of S100A8 in skin (*n* = 4) and heart (*n* = 7 or 8) of young and old cynomolgus monkeys with higher magnification of indicated region shown at right. Scale bars, 25 μm and 5 μm (zoomed-in image). Data are shown as means ± SEM. *n*, number of animals, ***P* < 0.01, (*t* test). (D) Representative immunohistochemistry images of S100A9 in skin (*n* = 6) and heart (*n* = 7 or 8) of young and old cynomolgus monkeys with higher magnification of indicated region shown at right. Scale bars, 25 μm and 5 μm (zoomed-in image). Data are shown as means ± SEM. *n*, number of animals. ***P* < 0.01, (*t* test). (E) Immunofluorescence staining of Ki67 of hAECs after co-treatment with rhS100A8/S100A9 (2 μg/mL) and H_2_O_2_ (100 μM/L). Data are shown as means ± SEM. Scale bars, 20 μm. *n* = 3, biological replicates. ns, non-significant, **P* < 0.05, ***P* < 0.01, (*t* test).

**Supplemental table legends**

**Table S1** The affinity score of genes encoding secreted proteins across *Homo sapiens*, *Macaca fascicularis*, *Rattus norvegicus* and *Mus musculus*.

**Table S2** Information of the serum donors used in this paper.

**Table S3** The RT-qPCR primers used in this paper.
